# Tribune Items

**Published:** 1874-12

**Authors:** 


Tribune Items.—We get a great many good items on health topics out of the *Tribune* about these days. In fact, that great daily is nearly as valuable to literary and scientific persons as to the news gleaner. Its literary and scientific notes are always entertaining and trustworthy.

